# Use of *Spondias Mombin* fruit pulp as a substrate for biosurfactant production.

**DOI:** 10.1080/21655979.2020.1853391

**Published:** 2020-12-21

**Authors:** Kenneth Ejike Ogbonna, Chidozie Victor Agu, Christopher Chukwudi Okonkwo, Kingsley Tochukwu Ughamba, Joseph Akor, Obioma U. Njoku

**Affiliations:** a Department of Science Laboratory Technology, Faculty of Physical Sciences, University of Nigeria, Nsukka, Nigeria; b Department of Biochemistry, Faculty of Biological Sciences, University of Nigeria, Nsukka, Nigeria; c INanoBio Inc., Scottsdale, AZ, USA; d Department of Animal Sciences, The Ohio State University, and Ohio State Agricultural Research and Development Center (OARDC), Wooster, OH, USA; e Department of Microbiology, Faculty of Biological Sciences, University of Nigeria, Nsukka, Nigeria

**Keywords:** Biosurfactants, *spondias mombin*, *Pseudomonas* spp, emulsification index

## Abstract

In this study, we explored the possibility of utilizing the succulent pulp of *Spondias mombin* (SM) as feedstock for the synthesis of biosurfactants by *Pseudomonas* spp. The cultures were composed of basic mineral medium amended with SM, SM + glucose, glucose (GLC), and nutrient broth (NB) as carbon sources. Biosurfactant production was determined by surface-active properties such as hemolysis, emulsification index (E_24_), drop collapse, oil-spreading assays, and reduction of surface tension. The stability of the biosurfactants was monitored across different temperature and pH regimes while chemical components of the extracted biosurfactants were determined by thin-layer chromatography. Biosurfactants synthesized from SM as sole substrate showed the highest emulsification index (56.35%), oil-spreading capacity (4.4 ± 1.31 cm), hemolysis (3.10 ± 0.02 cm), the shortest time for drop collapse (30 s), and surface tension reduction (24 mN/m). Biosurfactant concentrations ranged from 0.07 ± 0.01 in the NB to 2.08 ± 0.01 g/L in the media amended with SM. Chemical characterization revealed significant concentrations of carbohydrates and lipids in the biosurfactant produced from SM (1.2 ± 0.17 and 0.88 ± 0.04 g/L, respectively) when compared to SM + glucose (0.92 ± 0.05, and 0.62 ± 0.02 g/L, respectively), glucose (0.35 ± 0.04 and 0.13 ± 0.02 g/L, respectively), and nutrient broth (0.06 ± 0.03 and 0.01 ± 0.01 g/L, respectively). The biosurfactants were stable over a wide range of temperature while E_24_ increased with pH. Our results show the viability of SM fruit pulp as low-cost feedstock for industrial-scale production of biosurfactants using *Pseudomonas* spp.

## 1. Introduction

**Figure:**
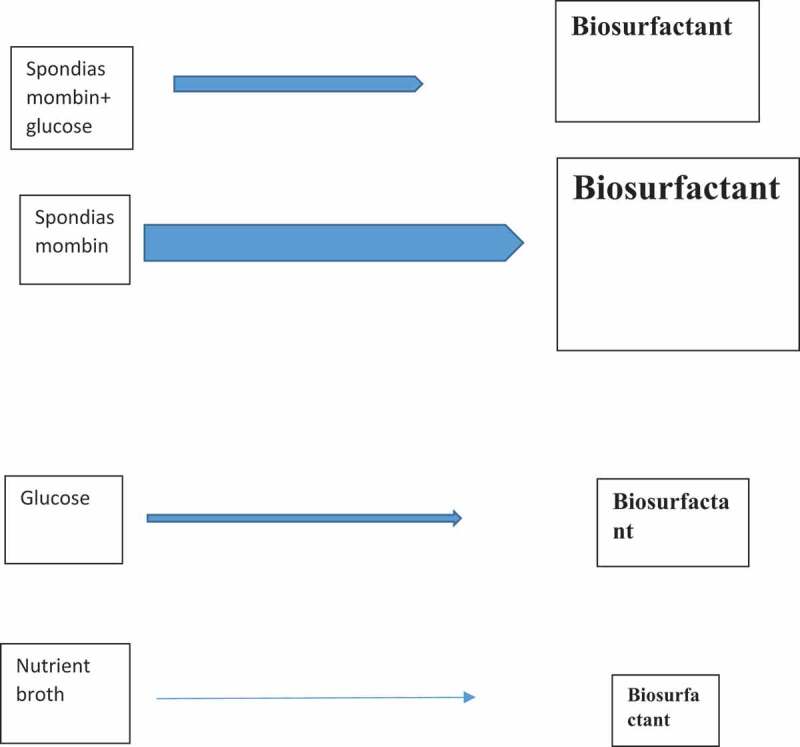

A major challenge with microbial production of feedstock chemicals is the cost of substrates. Most fermenting microbes prefer glucose as the primary fermenting substrate. Unfortunately, glucose is mainly produced from food-based sources and its use as industrial feedstock increases the price of animal feed, further fueling the food versus fuel debate. There are concerted research efforts aimed at exploring non-food-based agro-industrial wastes as alternative feedstocks to produce biofuels and chemicals via fermentation. In line with this global effort, the overarching goal of this study is to explore less fashionable agro-based feedstocks such as *Spondias mombin* fruit as an alternative to food-based substrates for fermenting microorganisms. *Spondias mombin* is a wild tree that is common in southern parts of Nigeria. The fruit, which is rich in carbohydrates and lipids, are hardly harvested and often left to rot as waste [1]. The goal of this study is to valorize *Spondias mombin* fruit pulp as an important substrate for the industrial production of feedstock chemicals including biosurfactants.

Microbially produced surfactants (biosurfactants) are surface-active amphiphilic molecules consisting of a structurally diverse group of biomolecules including glycolipids, lipopeptides, lipoproteins, lipopolysaccharides, and phospholipids [2]. They are accumulated by microbial cells or released in the extracellular environment by some microorganisms when growing on predominantly water immiscible substrates [3]. The ability of biosurfactants to lower surface tensions at the phase boundary between liquids and substrates ensures nutrient accessibility by these microorganisms, enabling nutrient uptake and metabolism, and allowing growth on water-immiscible substrates. Generally, surfactants possess highly emulsifying, foaming, and dispersing abilities [4]. They are applied in enhanced oil recovery, food processing, cosmetic, and pharmaceutical industries. In medicine, they are used as immunological adjuvants, antimicrobial, anticancer and anti-adhesive agents, and gene delivery [5]. Surfactants are important for bioremediation processes – the use of microorganisms to breakdown soil and water pollutants – for decontamination of the environment [6]. Bioremediation is often performed in tandem with bio-stimulation, which is the addition of nutrients to enhance the biological removal of contaminants by hydrocarbon-degrading bacteria [7]. The addition of surfactants increases the hydrocarbon bioavailability and enhances the growth of microorganisms thereby improving the breakdown of the pollutants [1]. Similarly, supplementation of heavy metal-contaminated soils with surfactants leads to the formation of surfactant-heavy metal complexes within the soil surface. Formation of the complex is accompanied by assimilation of these metals by heavy metal-accumulating bacteria or plant [8].

Unfortunately, many surfactants used today are produced chemically and pose serious environmental and toxicological challenges due to their recalcitrance [7,8]. To overcome the toxic and environmental impact of synthetic surfactants, research efforts are directed toward green technology – the use of cheap agricultural wastes for microbial synthesis of biosurfactants [9]. The advantages of microbial surfactants include biodegradability, low toxicity (or higher EC 50 which is the concentration that can effectively decrease 50% of the test population), biocompatibility, and digestibility which make them possible to be applied in food industries as additives, cosmetics, and pharmaceuticals. Further, the availability of cheap agro-wastes feedstocks, e.g.,, *Spondias mombin* fruits adds value to large-scale production of biosurfactants via microbial fermentation [9]. Importantly, *Pseudomonas* species, an established metabolic workhorse for microbial production of biosurfactants can utilize alternative low-cost substrates for fermentation [10–13]. In the current research, we show the potentials of *Spondias mombin* fruit waste as viable feedstock to produce biosurfactants by *Pseudomonas* species. To the best of our knowledge, this is the first attempt at using *Spondias mombin* fruit as a carbon source for the microbial production of any feedstock chemical.

## 2. Materials and methods

### 2.1 Preparation of plant material

The pulp of freshly collected *Spondias mombin* (SM) fruits was carefully separated from the seeds. An optimized quantity (147 g) of the fruit pulp was extracted with 250 mL of methanol. The methanol SM extract was dried in an oven set at low heat, 50°C for 24 h. The dry powder was stored in an air-tight container and subsequently used as a carbon source for the preparation of *Pseudomonas* spp growth media. To identify the active constituents of the fruit, qualitative phytochemical analysis of the fresh and dry SM samples was performed following a standard protocol [14]. The AOAC method [15] was used to determine the proximate constituents.

### 2.2 Microorganism and culture conditions

Laboratory stock of *Pseudomonas* spp slant culture was re-activated using established protocol [16]. In a sterile hood, the slant culture was streaked on nutrient agar and then incubated at 25°C for 24 h to allow the formation of single colonies. The preculture was prepared by inoculating a single colony of *Pseudomonas* spp into 100 mL of nutrient broth (Sigma Aldrich, St Louis, MO) followed by incubation at a temperature of 25°C and 150 rpm for 6 h. The basal mineral medium (BMM) consisted of 12.5 g/L K_2_HPO_4_, 3.8 g/L KH_2_PO_4_, 1 g/L (NH_4_)_2_SO_4_, 0.1 g/L MgSO_4_.7H_2_O, and 5 ml of trace element solution containing 0.232 g/L H_3_BO_3_, 0.174 g/L ZnSO_4_.7H_2_O, 0.116 g/L FeSO_4_, 0.096 g/L CoSO_4_.7H_2_O, 0.022 g/L (NH_4_)_6_Mo_7_O_24_.4H_2_O, 8.0 mg/L CuSO_4_.5H_2_O, and 8.0 mg/L MnSO_4_.4H_2_O. Four basal mineral media (BMM) containing different carbon sources were prepared as follows: (1) 14.4 g/L SM only, (2) 7.2 g/L each of SM and glucose, (3) 14.4 g/L glucose only, and (4) 14.4 g/L nutrient broth. Media were autoclaved at 121°C for 15 min. Fermentation was carried out in loosely capped 250-mL Pyrex culture bottles using 6% (v/v) of *Pseudomonas* spp pre-culture in 50 ml of culture medium (basal mineral medium + carbon source). All cultures were grown in triplicate at 32 ± 1°C and 150 rpm for 12 days. BMM without carbon source served as the negative control. Samples were collected every 24 h to determine the growth of the cells and to analyze biosurfactant production and activity.

### 2.3 Isolation and purification of biosurfactants

Two ml culture was centrifuged at 10,000 x g for 2 min to remove cell pellets. Biosurfactants were isolated from the cell-free supernatant using the acid precipitation method by adjusting the pH to 2.0 using 6 N HCl [17]. Afterward, the extract was incubated overnight at 4°C. The precipitate formed was collected by centrifugation at 10,000 rpm and 4°C for 20 min. This crude extract was then applied to column chromatography on reverse phase silica gel with a mesh size of 60–120 and eluted in a stepwise manner using 100% methanol at a flow rate of 1 mL/min at 25°C. The amount of biosurfactant produced was measured by gravimetry and expressed in mg per mL of culture used for extraction.

### 2.4 Screening of culture supernatants for biosurfactant activity

Biosurfactants consisted of a structurally different groups of biomolecules including glycolipids, lipopeptides, lipoproteins, lipopolysaccharides, or phospholipids [2]. Consequently, the methods used to screen biosurfactant activity depends on the physical effects of surfactants. Standard surfactant activity tests based on surface/interfacial tension were used to determine the presence of biosurfactants produced by *Pseudomonas* spp. These include hemolysis, oil drop collapse, emulsification capacity, oil-spreading assays, and surface tension measurement.

### 2.4.1. Hemolytic assay

The hemolysis assay was performed following the method of Mulligan et al. [18]. Biosurfactant-producing cells will break erythrocytes leading to the formation of a colorless and transparent ring around the colony. Blood agar was prepared by dissolving 2.4 g of nutrient agar in 100 mL of distilled water. The mixture was sterilized at 121°C for 15 min and then allowed to cool followed by aseptic addition of 5 mL of fresh sheep blood. The blood agar was distributed into sterile petri dishes and allowed to solidify. Next, a loopful of culture was used to streak the solidified blood agar, followed by incubation at 25°C for 24 h. The plates were incubated and examined to locate zones of clearance. Diameters of zones of clearance due to hemolysis around colonies on the agar plates was measured to the nearest millimeter (mm) using a transparent meter rule.

### 2.4.2. Drop collapse assay

The assay principle involves using surfactants to destabilize oil droplets. Drop collapse assay was done following the method described by Khan et al. [19]: Briefly, 20 µl of crude oil was added into wells of a 96-well plate. The plate was equilibrated at 25°C for 1 h after which 50 µl of culture supernatant was added. The shape of the oil drop was observed after 1 min. The collapse of the surface of crude oil by the culture supernatant is indicative of a positive result. If the drop remained (did not collapse), it indicates a negative result. About 1 mg/mL Tween-80 solution was used as a standard (positive control).

### 2.4.3. Oil spreading/Displacement assay

This test was done as previously described [20]. To make a thin oil layer, 20 mL of crude oil was added to 40 µl of distilled water contained in a petri dish. Next, 10 µl of supernatant was then added to the center of the oil. The presence of biosurfactant will lead to the formation of an oil-free clear zone. The diameter of the zone of clearance positively correlates with biosurfactant activity. The negative control contained 10 µl plain growth medium instead of culture supernatant, while positive control composed of 10 µl Tween-80.

### 2.4.4. Emulsification capacity assay

This assay was performed following the protocol described by Kumari et al. [21]. Cell-free supernatants (CFS) were prepared by centrifuging incubated cultures at 10, 000 × g for 20 min. One mL of kerosene, crude oil, or olive oil was added to an equal volume of CFS followed by agitation on a vortex at high speed for 2 min. To measure the emulsification capacity, the mixture was made to stand for 24 h after which the height of the emulsion layer was determined. The emulsification index (E_24_) is defined as the percentage of the height of the emulsified layer (in cm) divided by the total height of the liquid column (in cm) [22].

$$E_{24}=\frac{Height\;of\;Emulsion\;layer\;x100\%}{Total\;height}$$

### 2.4.5. Temperature and pH stability tests

To determine the thermal stability, culture supernatants were subjected to varying temperatures (30°C to 90°C) for 1 h and then cooled to 25°C. After subjecting to each temperature, the E_24_ value of biosurfactants in the culture supernatant was measured. Similarly, the pH stability was measured by adjustment of pH from 2 to 12 using 1 M HCl or NaOH, followed by determination of E_24_.

### 2.4.6. Surface tension measurement

A KRUSS KIOT Tensiometer (KRUSS, Optische–Mechanische Werkstatten, Hamburg, Germany) equipped with a 6 cm De Nuoy platinum ring was used to measure the surface tension of the culture broth. During the study, an average of triplicate samples was used in other to increase accuracy.

### 2.4.7. Characterization of biosurfactant by thin-layer chromatography

The different biosurfactants produced were characterized following the thin-layer chromatography method [19,23]. Briefly, 0.1 g quantity of each of the purified isolated biosurfactant samples was dissolved in methanol and analyzed qualitatively by thin-layer chromatography (TLC) using plates of silica gel. After air-drying, chromatograms were developed with a mix of chloroform:methanol: acetic acid in 65:15:2 ratio (v/v) as the mobile phase and detected using the following protocol: for sugar detection, exposure to Molish reagent; for lipid detection, exposure to iodine vapor; for amino acid determination, 1% ninhydrin solution was applied. Following exposure, the TLC plates were subjected to heat at 110°C for 40–45 min for color development.

### 2.5 Statistical analysis

All data were reported as mean ± standard deviation of triplicate determinations. The general linear model of Minitab version 17 (Minitab Inc., State College, PA, USA) was used for all statistical analyses to compare the differences between carbon sources as they pertain to biosurfactants, carbohydrates, and lipids concentrations, hemolysis, oil-spreading activity, surface tension measurement, and emulsification indices. Significant differences were established at p ≤ 0.05.

## 3. Results

### 3.1. *Phytochemical and proximate analyses of* Spondias mombin *pulp extract*

Phytochemical and proximate analyses were conducted to determine the bioactive components of SM. The qualitative data indicate that flavonoids, proteins, and steroids were present in moderate quantities (Table 1). In addition, carbohydrates, glycosides, reducing sugars, and oils occurred in larger amounts whereas saponins and alkaloids were only present in minute quantities (Table 1). Tannins and terpenoids were undetected in SM-pulp extract. The quantitative data shows that alkaloids, flavonoids, and steroids were present at 0.07, 0.16, and 0.79 mg/g of SM-pulp extract, respectively, and were reflective of the qualitative data. Further, proximate analysis of SM-pulp extract shows a high percentage of moisture (88.48%) and carbohydrates (5.30%). The other proximate content includes fats (2.54%), proteins (1.40%), ash (1.6%), and fiber (0.67%).

**Table 1.** Phytochemical and proximate composition of *Spondias mombin* pulp extract

Phytochemicals	Abundance	Proximate parameter	Percentage (%)
Alkaloids	+	Moisture	88.48
Flavonoids	++	Proteins	1.40
Glycosides	+++	Fats	2.54
Proteins	++	Fiber	0.67
Carbohydrates	+++	Ash	1.59
Saponins	+	Carbohydrate	5.30
Steroids	++		
Reducing sugars	+++		
Tannins	-		
Terpenoids	-		

Key: +++ present in high amount, ++ present in moderate amount, + present in low amount,- absent.

### 3.2. Cell growth in different media

**Figure 1.**
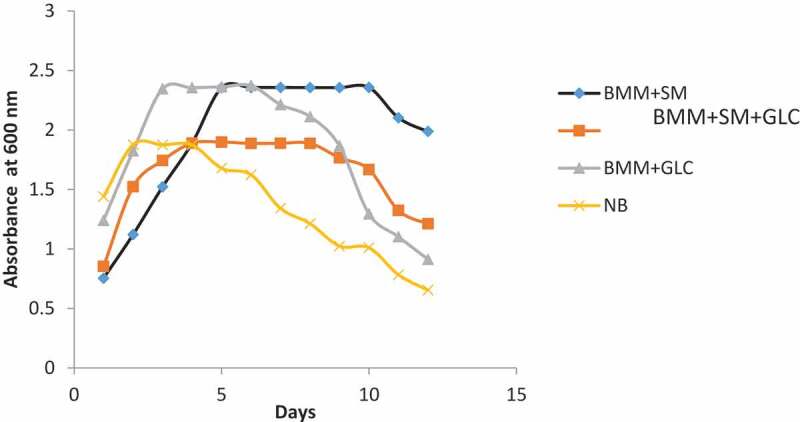
The growth pattern of *Pseudomonas species* in the different media

The growth pattern of *Pseudomonas* in the different media was determined by measuring culture optical density for a 12-day period. As shown in Figure 1, basal mineral medium supplemented with SM supported the growth of *Pseudomonas* spp for up to 10 days after which a decline in growth was observed. The maximum cell growth for the SM-only culture was ~1.6-fold higher than in the nutrient broth (NB) culture. However, the maximum growth of *Pseudomonas* spp in SM-only medium mirrored that observed for the glucose-only medium. Nonetheless, the SM-only medium supported an extended growth of *Pseudomonas* spp as indicated in the prolonged stationary growth phase observed in the SM-only medium relative to the glucose-only medium (Figure 1). Similarly, the growth of *Pseudomonas* spp in SM supplemented with glucose (SM+GLC) medium also had a prolonged stationary growth phase (Figure 1). The observed-extended stationary growth phase of *Pseudomonas* spp in media containing SM when compared to GLC-only medium suggests that SM may be a suitable substrate for *Pseudomonas* spp cultivation and for biosurfactant production.

### 3.3. Biosurfactant concentration in different media

The amount of biosurfactants synthesized using *Pseudomonas* spp in different media is presented in Table 2. *Pseudomonas* spp produced the most and least amounts of biosurfactants from SM and NB media, respectively, while no biosurfactant was synthesized in the basal mineral medium without any carbon source (Table 2). Interestingly, biosurfactant production in the SM and SM+GLC media increased 4.3- and 3.2-fold, respectively, relative to the GLC-only medium (Table 2). Similarly, the concentrations of biosurfactant in the SM and SM+GLC media were 29.7- and 22.0-fold of those recorded in the NB medium suggesting that *Pseudomonas* spp may have preferred SM as a substrate for biosurfactant production (Table 2).

**Table 2.** The concentration of biosurfactants in different media

Carbon source	Biosurfactants (g/L)
BMM+SM	2.08 ± 0.01^a^
BMM+SM+GLC	1.54 ± 0.02^b^
BMM+GLC	0.48 ± 0.04 ^c^
BMM+NB	0.07 ± 0.01^d^
BMM	0.00

BMM = Basal mineral medium; SM = *Spondias mombin* pulp extract.

^a,b,c,d^Fisher’s LSD pairwise Values with different letters indicate significant difference at p < 0.05.

### 3.4. Biosurfactant activity analysis

The biosurfactants produced from the different carbon sources evaluated were characterized for their activities via hemolysis, drop collapse, oil spreading, and surface tension measurement tests. As shown in Table 3, the hemolytic activity of the biosurfactant synthesized from SM substrate had a larger clearing zone with a diameter of 3.10 cm. This value was ~2.1-fold higher than the clear zone observed for the biosurfactant produced from GLC-only substrate (Table 3). Likewise, the biosurfactant produced from the SM+GLC substrate gave a hemolytic clear zone of 2.51 cm and corresponds to a 1.67-fold increase in the hemolytic clear zone when compared to those observed for the biosurfactants synthesized from the GLC-only substrate (Table 3).

**Table 3.** Comparison of biosurfactants activities among carbon sources in hemolytic, drop collapse, oil spreading, and surface tension reduction tests

Carbon source	Hemolytic test (cm)	Drop assay	Time of collapse (s)	Oil spreading test (cm)	Surface tension measurement (mN/m)
SM	3.10 ± 0.02^a^	+++	30	4.4 ± 1.31^a^	24 ± 0.02^a^
SM+GLC	2.51 ± 0.02^b^	++	60	2.73 ± 0.06^b^	27 ± 0.05^b^
GLC	1.5 ± 0.01 ^c^	++	150	1.64 ± 0.05 ^c^	29.2 ± 0.01 ^c^
NB	0.9 ± 0.02^d^	+	150	0.97 ± 0.05^d^	32 ± 0.04^d^
Tween 80	3.8 ± 0.02^e^	+++	30	15.2 ± 0.1^e^	25 ± 0.03^e^

SM = *Spondias mombin* pulp extract.

^a,b,c,d,e^Fisher’s LSD pairwise Values with different letters indicate significant difference at p ≤ 0.05. Drop collapse assay: +++ present in high amount, ++ present in moderate amount, + present in low amount.

Furthermore, the oil drop collapse times for the biosurfactants produced from SM and SM+GLC substrates decreased 5.0- and 2.5-fold relative to the biosurfactant produced from GLC-only substrate (Table 3). This shows that the biosurfactant produced from SM destabilizes oil relatively faster than those produced from GLC-only substrate. In addition, the oil-spreading assay revealed that the clearing zone diameters of the biosurfactants from SM and SM+GLC substrates were 2.7- and 1.7-fold higher when compared to the clearing zone diameters of the biosurfactant synthesized from GLC-only medium. The surface tension reduction ability of the biosurfactants was 1.2- and 1.1-fold lower relative to those produced from GLC-only medium (Table 3).

### 3.5. Biosurfactant emulsification indices on oils, diverse temperatures, and pH

**Figure 2.**
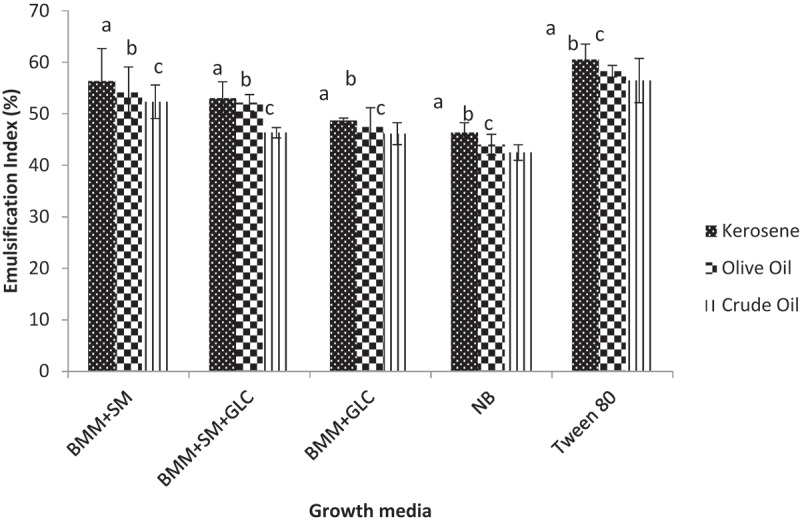
The emulsification patterns on different oils using different growth media

The emulsification patterns of biosurfactants synthesized from different growth media were evaluated using oils and the data are presented in Figure 2. The emulsification indices (E_24_) of biosurfactants on oils ranged from 43% in NB to 56.35% in SM media. The highest E_24_ value was obtained with kerosene whereas the least was recorded with crude oil (Figure 2). The E_24_ of SM biosurfactant in kerosene (56.35%) was comparable to the E_24_ obtained with Tween-80 (60%). The E_24_ of SM biosurfactant in kerosene was ~1.1-, 1.2-, and 1.2-fold higher than the E_24_ of SM+GLC (53%), GLC (48%), and NB (46.35%) biosurfactants, respectively. Similar emulsification patterns were observed with crude and olive oils (Figure 2).

**Figure 3.**
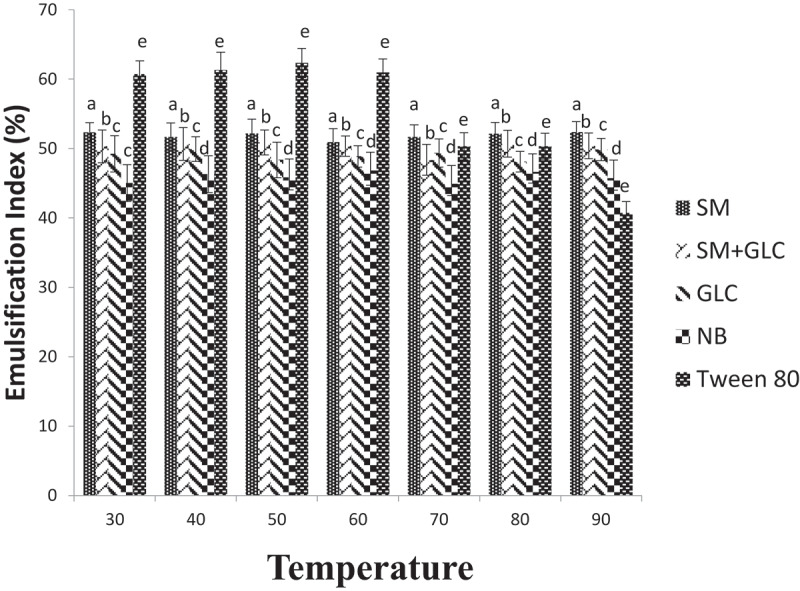
Emulsification index in different growth media under different temperature regimes

The E_24_ values of biosurfactants produced from SM, SM+GLC, GLC, NB, and the E_24_ values of Tween 80 (standard biosurfactant) at different temperatures are presented in Figure 3. The biosurfactant produced from SM had the highest E_24_ value and remained relatively unchanged at all the temperatures (30–90°C) tested. Temperature change appears to have no significant impact on the E_24_ values of biosurfactants synthesized from SM+GLC, GLC, and NB substrates.

**Figure 4.**
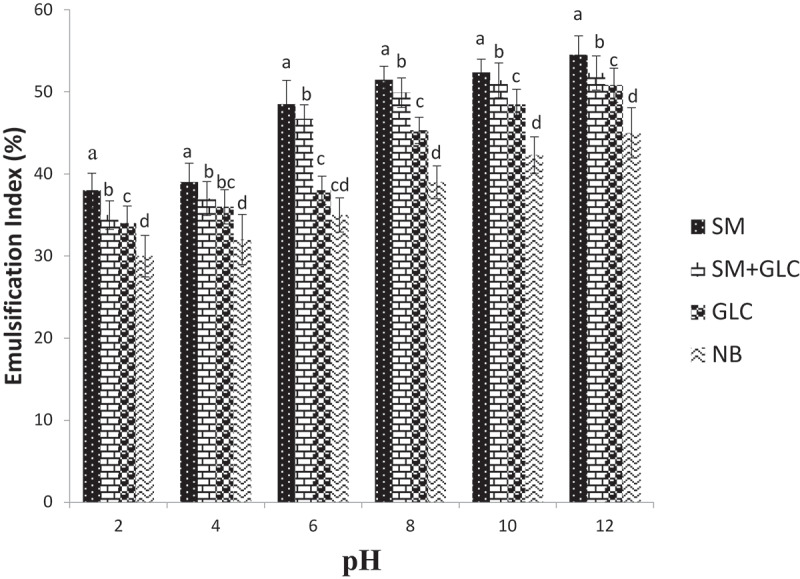
Emulsification index in different growth media under different pH regimes

The E_24_ of biosurfactants in the pH regime test showed that biosurfactants produced from SM substrate had the highest values and ranged from 38% at pH 2 to 56% at pH 12 (Figure 4). Biosurfactants synthesized in the NB substrate had the least E_24_ values and ranged between 28% at pH 2 and 46% at pH 12 (Figure 4). Further, at pH 6 the biosurfactants produced in the SM and SM+GLC substrates had E_24_ values that are ~1.3- and 1.2-fold, respectively, greater than those produced from GLC-only substrate at all the pH evaluated (Figure 4).

BMM = Basal mineral medium.SM = *Spondias mombin* pulp extract.GLC = Glucose.NB = Nutrient broth.

### 3.6. Characterization of biosurfactants

The biosurfactants produced in the media containing different carbon sources were characterized by TLC. Carbohydrates and lipids were shown to be abundant in the biosurfactants whereas proteins remained undetected (Table 4). The carbohydrate and lipid content of the biosurfactants were in the range of 1.2 and 0.88 to 0.06 and 0.01 g/L, respectively (Table 4). In addition, biosurfactants from SM, SM+GLC, GLC, and NB had carbohydrates to lipids ratio of 1.4:1, 1.5:1, 2.7:1, 6:1, respectively (Table 4). Interestingly, the observed pattern suggests that biosurfactants with higher lipid content may possess better biosurfactant properties.

**Table 4.** Comparison of carbohydrate and lipid concentrations among carbon sources

Carbon source	Proteins (g/L)	Carbohydrates (g/L)	Lipids (g/L)
SM	Not detected	1.20 ± 0.17^a^	0.88 ± 0.04^a^
SM+GLC	Not detected	0.92 ± 0.05^b^	0.62 ± 0.02^b^
GLC	Not detected	0.35 ± 0.04 ^c^	0.13 ± 0.02 ^c^
NB	Not detected	0.06 ± 0.03^d^	0.01 ± 0.01^d^

^a,b,c,d^Fisher’s LSD pairwise Values with different letters indicate significant difference at p < 0.05.

## 4. Discussion

Previous studies on *Pseudomonas* species were centered on their pathogenicity, medical importance, and biosurfactant production from different soluble and insoluble carbon sources, including glucose, trehalose, sucrose, fructose, maltose, glycerol, palmitic acids, soybean oil, corn oil, canola oil, olive oil, long-chain n-alkanes, and 1-hexadecane [24,25]. In the current study, we have focussed on the production of biosurfactants using *Spondias mombin* agricultural waste. The *S. mombin* plant is considered a wild plant in southern Nigeria; hence, its fruits are not consumed often, leading to the generation of a significant amount of agricultural wastes. In line with global efforts to reduce the impact of wastes on the environment and promote green chemistry, we explored the production of biosurfactants by *Pseudomonas* spp. using the pulp extract of SM fruit as a carbon source. Interestingly, it was deduced that SM-pulp is rich in nutrients (Table 1), hence suitable as a substrate for biosurfactant production by *Pseudomonas* spp. The considerable amount of carbohydrates, proteins, and lipids shows that SM-pulp extract is a suitable substrate for fermentation.

While the maximum absorbance in cultures grown on SM and GLC substrates was comparable (2.40), cultures grown in SM had a longer stationary phase (Figure 1). The longer stationary phase probably enhanced biosurfactant production. Biosurfactants are produced as secondary metabolites due to stress encountered in the utilization of complex substrates. Substrate complexity may become a stressor to biosurfactant-producing microorganisms and when such stressors are encountered biosurfactants are synthesized to enable emulsification and subsequent catabolism of the complex substrates for absorption. The *S. mombin* may have posed greater stress to *Pseudomonas* spp that probably resulted in enhanced biosurfactant production relative to when glucose or nutrient broth were supplied as a source of carbon. Biosurfactants produced from the four substrates varied significantly (p ≤ 0.05; Table 2). The variance in yields may be attributed to the nutritional environment, such as the concentration of nitrogen, phosphorus, magnesium, ferric ion, manganese ions, as well as the nature of carbon substrate in the growth medium of *Pseudomonas* spp. *Spondias mombin* contains different sugar types which were utilized by *Pseudomonas* spp. The ability of *Pseudomonas* spp to metabolize mixed sugars is of paramount importance in the use of inexpensive agricultural wastes as fermentation substrates for the production of biosurfactants and other value-added industrial products. The variation in biosurfactant yields from different substrates corroborates the study by Mouafo et al. [9] where they noted that biosurfactant production varied significantly (p ≤ 0.05) among organisms and substrates.

The biosurfactant produced from SM-pulp extract by *Pseudomonas* spp showed superior characteristics. For instance, the SM biosurfactant exhibited a significant (p < 0.05) hemolysis clearing zone (Table 3), indicating its effectiveness as a potent biosurfactant. In addition, SM and SM+GLC biosurfactants collapsed oil within 30 and 60 s, which corresponds, respectively, to a 5- and 2.5-fold decrease in oil collapse times relative to the GLC or NB biosurfactant (Table 3). In a similar study by Thavasi et al. [26], biosurfactant produced from crude oil collapsed oil at 60 s. Furthermore, the oil-spreading activities of SM and SM+GLC biosurfactants were higher than what was observed with the GLC and NB biosurfactants (Table 3). Biosurfactants synthesized by *Pseudomonas aeruginosa* have been reported to cause oil-spreading diameter of 1.5 cm [27]. However, Thavasi et al. [26] showed that biosurfactants produced from crude oil by *P. aeruginosa* caused oil-spreading diameter of 3.5 cm. In our study, the SM biosurfactant produced by *Pseudomonas* spp had a higher oil-spreading diameter of 4.40 cm indicating that *Spondias mombin* is a suitable substrate for biosurfactants production. The surface tension reduction ability of the biosurfactants produced from the diverse substrates tested ranged from 24 to 32 mN/m. These results were comparable to the previous report [28]. The SM biosurfactant exhibited a significant (p < 0.05) reduction in surface tension and could indicate that SM biosurfactants may be composed of rhamnolipids. Rhamnolipids are known to display a significant reduction in surface tension [29]. However, NMR and mass spectroscopy analyses of these biosurfactants produced by *Pseudomonas* spp would shed more light.

Emulsification of oils has been widely used to assay for the presence and concentration of biosurfactants [9,30]. In the current study, an emulsification test was carried out with kerosene, olive oil, and crude oil. There were significantly higher (p ≤ 0.05) emulsification indices (E_24_) recorded with SM biosurfactant. The least emulsification index was observed with NB biosurfactant. Furthermore, it was noted that E_24_ among the different hydrocarbons followed the order: kerosene> olive oil > crude oil (Figure 2). Measurement of emulsification properties of surfactants is based on their emulsification activity (EA) and emulsification stability (ES). EA is the ability of a surfactant to form an emulsion which is inversely related to the size of the oil droplet (the smaller the size, the greater the activity). It stands to reason, therefore, that kerosene with the smallest oil droplet size had the highest emulsification index with biosurfactants (Figure 2). Priya and Usharani [27] revealed that biosurfactants produced by *Pseudomonas aeruginosa* using vegetable oil, petrol, and diesel as carbon sources had an emulsification index of 49% with kerosene. However, a higher biosurfactant activity with SM and SM + GLC biosurfactants were recorded in the present study (Figure 2). This was because of high concentrations of biosurfactants produced from these substrates (SM and SM+GLC) resulting, in part, from the complex nature of *Spondias mombin* pulp extract. The emulsification index of each of the culture supernatants with the oils was proportional to the quantity of biosurfactants contained in each culture. Mouafo et al. [9] noted a similar trend and asserted that biosurfactant production varies with the substrates used among other factors.

In the present study, biosurfactants produced by *Pseudomonas* spp in each culture were thermostable. In all cultures, the emulsification index was not affected by an increase in temperature, showing that the biosurfactants were stable at all temperatures and were able to maintain their surface properties at elevated temperatures (Figure 3). Regardless, SM containing cultures (SM-only and SM+GLC) exhibited higher emulsification indices at all temperatures when compared to glucose and nutrient broth (Figure 3). The stability of biosurfactants in culture supernatants was also monitored across the pH range (2–12). Emulsification index generally correlates directly with pH, showing that the stability of biosurfactants was affected by pH (Figure 4). The emulsification abilities of all four biosurfactants were lower in acidic conditions than in alkaline conditions. At pH <6, the samples become more turbid due to partial precipitation of the biosurfactants. Consequently, the precipitated biosurfactants possibly led to a reduction in emulsification activities at low pH (Figure 4). On the contrary, at pH ≥6, emulsification activity increased due to improved biosurfactant dissolution. This is because, under an alkaline environment (high pH), secondary metabolites precipitate out of solution and allows the fatty acids of biosurfactant micelles to become more stable [31]. Overall, the SM and SM+GLC biosurfactants exhibited higher emulsification indices with improved stability when compared to the GLC or NB biosurfactant at all tested pH (pH 2–12; Figure 4). The improved stability of SM biosurfactant over pH 2–12 may be attributed, in part, to the high concentration of biosurfactant synthesized from SM substrate. In a broader sense, the unique properties of biosurfactants produced by *Pseudomonas* spp allow for their wide-scale applications in several bioremediation studies since the utilization of biosurfactants in different fields depends on biosurfactants stability at various temperatures and pH [31]. Moreover, the temperature stability of biosurfactants is an important factor in the food, pharmaceutical, and cosmetic industries since their applications involve heating at elevated temperatures to achieve sterilization [17].

The thin-layer chromatography analysis shows that biosurfactants synthesized by *Pseudomonas* spp contain carbohydrates and lipid molecules with carbohydrates being the major component (Table 4). This indicates that the biosurfactants may be classified as glycolipids. *Pseudomonas* spp is known to produce glycolipids from diverse substrates including olive oil, palmitic acid, glucose, glycerol, soybean oil, corn oil, canola oil among others [17,25,32]. However, it has also been shown to synthesize protein emulsifier only from 1-hexadecane, acetyl alcohol, and long-chain n-alkane substrates [24].

## 5. Conclusion

Based on the results obtained in this study, the yield of biosurfactant produced by *Pseudomonas* spp. using the methanol pulp extract of *Spondias mombin* as a carbon source was higher than those produced using other carbon sources. The quantity and chemical composition or nature of the biosurfactant produced were greatly influenced by the phytochemistry and proximate composition of *Spondias mombin* as well as the microorganism (*Pseudomonas* spp) involved in the production. Furthermore, the extracted biosurfactant was a glycolipid molecule. Therefore, we conclude that *Spondias mombin* pulp extract is a good raw material for biosurfactant (glycolipid) production by *Pseudomonas* spp used in the present study.

## Research highlights

- Biosurfactants were produced by *Pseudomonas* spp using *Spondias mombin* fruit pulp (SM) as a novel substrate.
- The quality of SM-produced biosurfactants is comparable to synthetic surfactants.
- The characteristics of SM-derived biosurfactants is comparable to those from glucose and other carbon sources.
